# Supplementary material for: Dynamic fibroblast–immune interactions shape recovery after brain injury
Source: Nature. 2025 Sep 3;646(8086):934–44. doi: 10.1038/s41586-025-09449-2 (PMC12545229; doi:10.1038/s41586-025-09449-2)
Supplement: Supplementary file 1 — Supplementary Figs. 1 and 2 [file 41586_2025_9449_MOESM1_ESM.pdf]

nature portfolio

https://doi.org/10.1038/s41586-025-09449-2

# Supplementary information

# Dynamic fibroblast–immune interactions shape recovery after brain injury

In the format provided by the authors and unedited

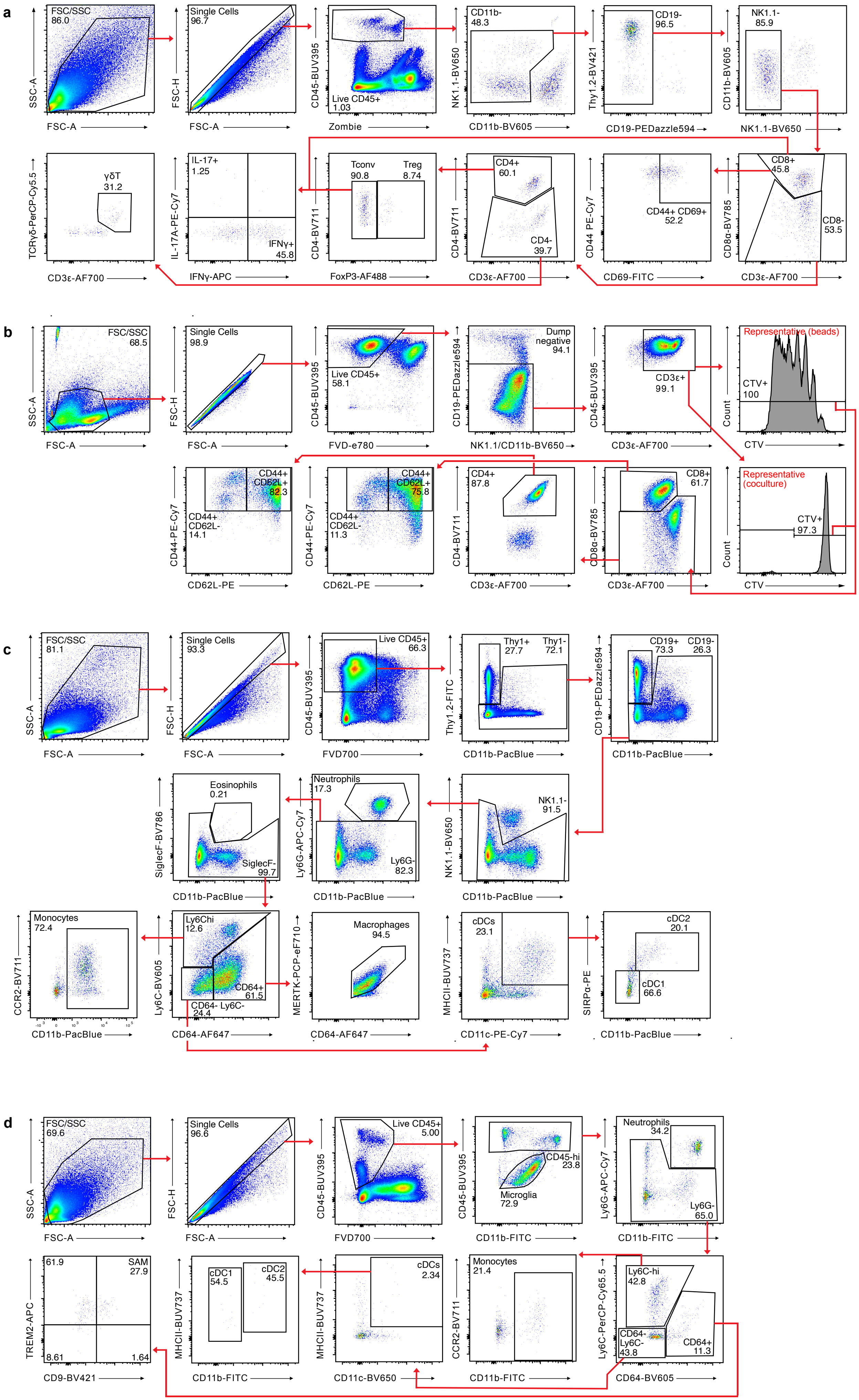

a
FSC/SSC 86.0
SSC-A
FSC-A
Single Cells 96.7
FSC-H
FSC-A
Live CD45+ 1.03
CD45-BUV395
Zombie
CD11b- 48.3
NK1.1-BV650
CD11b-BV605
CD19- 96.5
Thy1.2-BV421
CD19-PEDazzle594
NK1.1- 85.9
CD11b-BV605
NK1.1-BV650
γδT 31.2
TCRγδ-PerCP-Cy5.5
CD3ε-AF700
IL-17+ 1.25
IFNγ+ 45.8
IL-17A-PE-Cy7
IFNγ-APC
Tconv 90.8
Treg 8.74
CD4-BV711
FoxP3-AF488
CD4+ 60.1
CD4- 39.7
CD4-BV711
CD3ε-AF700
CD44+ CD69+ 52.2
CD44 PE-Cy7
CD69-FITC
CD8+ 45.8
CD8- 53.5
CD8α-BV785
CD3ε-AF700
b
FSC/SSC 68.5
SSC-A
FSC-A
Single Cells 98.9
FSC-H
FSC-A
Live CD45+ 58.1
CD45-BUV395
FVD-e780
Dump negative 94.1
CD19-PEDazzle594
NK1.1/CD11b-BV650
CD3ε+ 99.1
CD45-BUV395
CD3ε-AF700
Representative (beads)
CTV+ 100
Count
CTV
CD44+ CD62L+ 82.3
CD44+ CD62L- 14.1
CD44-PE-Cy7
CD62L-PE
CD44+ CD62L+ 75.8
CD44+ CD62L- 11.3
CD44-PE-Cy7
CD62L-PE
CD4+ 87.8
CD4-BV711
CD3ε-AF700
CD8+ 61.7
CD8α-BV785
CD3ε-AF700
Representative (coculture)
CTV+ 97.3
Count
CTV
c
FSC/SSC 81.1
SSC-A
FSC-A
Single Cells 93.3
FSC-H
FSC-A
Live CD45+ 66.3
CD45-BUV395
FVD700
Thy1+ 27.7
Thy1- 72.1
Thy1.2-FITC
CD11b-PacBlue
CD19+ 73.3
CD19- 26.3
CD19-PEDazzle594
CD11b-PacBlue
Eosinophils 0.21
SiglecF- 99.7
SiglecF-BV786
CD11b-PacBlue
Neutrophils 17.3
Ly6G- 82.3
Ly6G-APC-Cy7
CD11b-PacBlue
NK1.1- 91.5
NK1.1-BV650
CD11b-PacBlue
Monocytes 72.4
CCR2-BV711
CD11b-PacBlue
Ly6Chi 12.6
CD64+ 61.5
CD64- Ly6C- 24.4
Ly6C-BV605
CD64-AF647
Macrophages 94.5
MERTK-PCP-eF710
CD64-AF647
cDCs 23.1
MHCII-BUV737
CD11c-PE-Cy7
cDC2 20.1
cDC1 66.6
SIRPα-PE
CD11b-PacBlue
d
FSC/SSC 69.6
SSC-A
FSC-A
Single Cells 96.6
FSC-H
FSC-A
Live CD45+ 5.00
CD45-BUV395
FVD700
CD45-hi 23.8
Microglia 72.9
CD45-BUV395
CD11b-FITC
Neutrophils 34.2
Ly6G- 65.0
Ly6G-APC-Cy7
CD11b-FITC
61.9
SAM 27.9
8.61
1.64
TREM2-APC
CD9-BV421
cDC1 54.5
cDC2 45.5
MHCII-BUV737
CD11b-FITC
cDCs 2.34
MHCII-BUV737
CD11c-BV650
Monocytes 21.4
CCR2-BV711
CD11b-FITC
Ly6C-hi 42.8
CD64- Ly6C- 43.8
CD64+ 11.3
Ly6C-PerCP-Cy65.5
CD64-BV605

**Supplementary Fig. 1: Representative flow cytometry gating.**

**a**, Gating of T cells *in vivo* (cortex/meninges/blood), with resident memory T cell markers or cytokine expression (distinct panels with similar pregating. **b,** Gating of T cells in *ex vivo* coculture. **c,** Gating of myeloid cells in spleen. **d,** Gating of myeloid cells in other organs (cortex/meninges/blood).

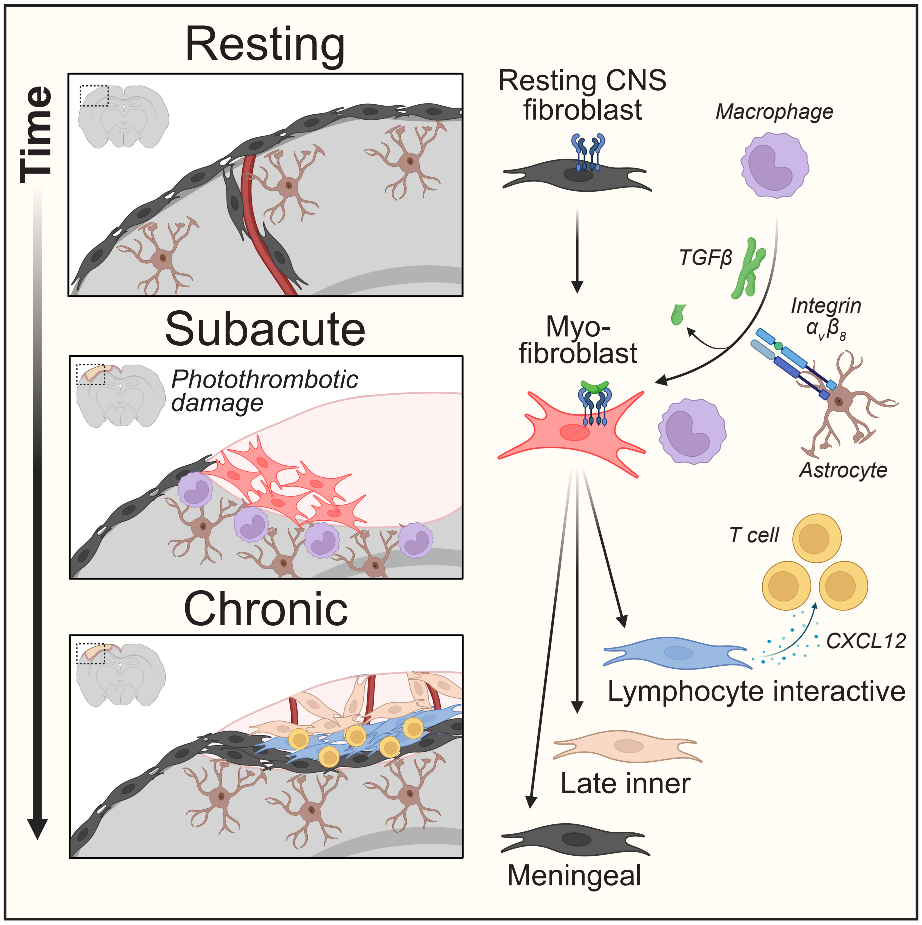


**Supplementary Fig. 2: Graphical Summary.**
