# Supplementary material for: Dynamic fibroblast–immune interactions shape recovery after brain injury
Source: Nature. 2025 Sep 3;646(8086):934–44. doi: 10.1038/s41586-025-09449-2 (PMC12545229; doi:10.1038/s41586-025-09449-2)
Supplement: Supplementary file 2 — Reporting Summary [file 41586_2025_9449_MOESM2_ESM.pdf]

nature portfolio

Corresponding author(s): Thomas D. Arnold
Ari B. Molofsky

Last updated by author(s): Jul 1, 2025

Reporting Summary

Nature Portfolio wishes to improve the reproducibility of the work that we publish. This form provides structure for consistency and transparency in reporting. For further information on Nature Portfolio policies, see our Editorial Policies and the Editorial Policy Checklist.

Statistics

For all statistical analyses, confirm that the following items are present in the figure legend, table legend, main text, or Methods section.

n/a	Confirmed	
☐	☒	The exact sample size (n) for each experimental group/condition, given as a discrete number and unit of measurement
☐	☒	A statement on whether measurements were taken from distinct samples or whether the same sample was measured repeatedly
☐	☒	The statistical test(s) used AND whether they are one- or two-sided<br>*Only common tests should be described solely by name; describe more complex techniques in the Methods section.*
☒	☐	A description of all covariates tested
☐	☒	A description of any assumptions or corrections, such as tests of normality and adjustment for multiple comparisons
☐	☒	A full description of the statistical parameters including central tendency (e.g. means) or other basic estimates (e.g. regression coefficient) AND variation (e.g. standard deviation) or associated estimates of uncertainty (e.g. confidence intervals)
☐	☒	For null hypothesis testing, the test statistic (e.g. F, t, r) with confidence intervals, effect sizes, degrees of freedom and P value noted<br>*Give P values as exact values whenever suitable.*
☒	☐	For Bayesian analysis, information on the choice of priors and Markov chain Monte Carlo settings
☒	☐	For hierarchical and complex designs, identification of the appropriate level for tests and full reporting of outcomes
☒	☐	Estimates of effect sizes (e.g. Cohen's d, Pearson's r), indicating how they were calculated

Our web collection on statistics for biologists contains articles on many of the points above.

Software and code

Policy information about availability of computer code

Data collection	Imaging data was collected using NIS-Elements v5.11.03 (Nikon), Zen Pro 2 v2.0.0.0 (Zeiss), or VS200 ASW v3.4.1 (Olympus). Flow cytometry data was collected using FACSDiva v9.0 (BD Biosciences). Pulse oximetry data was collected using the MouseOx Pulse Oximeter system (STARR Life Sciences). Blood pressure data was collected using the CODA-HT4 Noninvasive Blood Pressure System (Kent Scientific).
Data analysis	Images were analyzed with Imaris v9.8.0 (Oxford Instruments) or ImageJ v2.1.0/1.53c (NIH). Flow cytometry data was analyzed with FlowJo v10.7.2 (BD Biosciences). Graphs were created using Prism v10.1.1 (GraphPad Software, Inc).<br><br>Single cell RNA sequencing data was aligned to the mouse genome mm10 or Grcm39 using SpaceRanger v2.0.0 and CellRanger v7.1.0, v7.2.0, or v9.0.0 (10X Genomics). Further RNAseq analysis was performed using R version 4.3.2 in RStudio v2023.03.1, with Seurat version 4.2.1, 5.0.1, or 5.2.1. Additional packages used include Presto (v1.0.0), EnhancedVolcano (v1.20.0), Nebulosa (v1.12.0), ScCustomize (v2.0.1), clusterProfiler (v4.10.0), nichenetr (v2.0.5), spacexr (v2.2.1), monocle3 (v1.3.4), DESeq2 (v1.42.0), dplyr (v1.1.4), ply (v1.8.9), ape (v5.7-1), cowplot (v1.1.2), Matrix (v1.6-4), variancePartition (v1.32.2), MAST (v1.28.0), HGNChelper (v0.8.1), openxlsx (v4.2.5.2), RColorBrewer (v1.1-3), gridExtra (v2.3), ggpubr (v0.6.0), ComplexHeatmap (v2.18.0), tidyverse (v2.0.0), tibble (v3.2.1), biomaRt (v2.58.0), data.table (v1.14.10), glmGamPoi (v1.14.0), SeuratWrappers (v0.3.2), patchwork (v1.1.3), magrittr (v2.0.3), s2 (v1.1.6), gplots (v3.1.3), stringr (v1.5.1), ggnewscale (v0.4.9), ggbreak (v0.1.4), coin (v1.4-3), and dunn.test (v1.3.6). Custom code used for single cell RNA sequencing analysis is available at GitHub ("https://github.com/newingcrystal/CNS_Fibroblasts"). CellPhoneDB (cellphonedb package v4.1.0) utilized Python v3.11.0 in Jupyter Notebook.

For manuscripts utilizing custom algorithms or software that are central to the research but not yet described in published literature, software must be made available to editors and reviewers. We strongly encourage code deposition in a community repository (e.g. GitHub). See the Nature Portfolio guidelines for submitting code & software for further information.

Data

Policy information about availability of data

All manuscripts must include a data availability statement. This statement should provide the following information, where applicable:

- Accession codes, unique identifiers, or web links for publicly available datasets
- A description of any restrictions on data availability
- For clinical datasets or third party data, please ensure that the statement adheres to our policy

Murine spatial and single nuclear transcriptomic data generated in this paper are deposited in Gene Expression Omnibus (GEO) under the accession number GSE254164. Mouse genomes were downloaded via 10X Genomics, including Mm10 (https://cf.10xgenomics.com/supp/cell-exp/refdata-gex-mm10-2020-A.tar.gz) and Grcm39 (https://cf.10xgenomics.com/supp/cell-exp/refdata-gex-GRCm39-2024-A.tar.gz). The raw data from Boghdadi et al. (marmoset stroke) is available at GSE179141 (https://www.ncbi.nlm.nih.gov/geo/query/acc.cgi?acc=GSM5410579, Series 1). The raw data from Garza et al. (human TBI) is available at GSE209552 (https://www.ncbi.nlm.nih.gov/geo/query/acc.cgi?acc=GSE209552). The raw data from Jain et al. (human GBM) is available at GSE132825 (https://www.ncbi.nlm.nih.gov/geo/query/acc.cgi?acc=GSE132825). The raw data from Keren-Shaul et al., used to generate DAM scores, is available at GSE98971 (https://www.ncbi.nlm.nih.gov/geo/query/acc.cgi?acc=GSE98971, SubSeries GSE98969). The raw data from Yin et al., used to generate dysmaturity scores, is available at GSE239603 (https://www.ncbi.nlm.nih.gov/geo/query/acc.cgi?acc=GSE239603, SubSeries GSE234496). The data from Hobson et al., used to generate IFNg scores in neurons, is available at doi:10.1016/j.bbi.2023.04.008 (see Supplementary Table 1). The genes from Sbierski-Kind et al., used to generate TGFb/Myofibroblast scores, are supplied as Supplementary Table 1. The genes from Mroz et al., used to generate IFNg scores in myeloid cells, are supplied as Supplementary Table 2.

Research involving human participants, their data, or biological material

Policy information about studies with human participants or human data. See also policy information about sex, gender (identity/presentation), and sexual orientation and race, ethnicity and racism.

Reporting on sex and gender	Not applicable
Reporting on race, ethnicity, or other socially relevant groupings	Not applicable
Population characteristics	Not applicable
Recruitment	Not applicable
Ethics oversight	Not applicable

Note that full information on the approval of the study protocol must also be provided in the manuscript.

Field-specific reporting

Please select the one below that is the best fit for your research. If you are not sure, read the appropriate sections before making your selection.

☒ Life sciences ☐ Behavioural & social sciences ☐ Ecological, evolutionary & environmental sciences

For a reference copy of the document with all sections, see nature.com/documents/nr-reporting-summary-flat.pdf

Life sciences study design

All studies must disclose on these points even when the disclosure is negative.

Sample size	Sample sizes were estimated based on standard power calculations (a=0.05, 80% power) performed for similar published experiments. In general, statistical calculations were not used to predetermine sample sizes.
Data exclusions	All exclusions are noted in Methods and are based on pre--established criteria unless otherwise noted. For lesion size quantification, outliers were excluded if lesion diameter was visualized to be <25% of the average, representing technical errors during injury, resulting in a single exclusion (see Methods; established prior to analysis/unblinding). Mice with hydrocephalus were also excluded. Exclusion criteria for tMCAO survival analysis include death during surgery, cohorts with mice showing weight <=75% of expected at time of surgery (established based on pilot data), or cohorts with excessive bleeding. For serum analysis, severely hemolyzed samples were excluded according to pre-established ULAM and manufacturer guidelines.
Replication	Select experiments were performed once, for reasons including breeding, cost, and technical constraints, including the following: tMCAO in Cthrc1(creER); R26(TdT) mice; analysis of spinal cords of Tgfbr2 cKO mice; analysis of cortical scar-associated macrophages in Tgfbr2 cKO mice; meningeal fibroblast coculture (using fibroblasts sorted from 18 mice); quantification of recombination in Ng2creER mice; and quantification of Col1a1GFP+ fibroblasts after ADWA11 treatment. RNA sequencing experiments contained two pooled mice per sample. All other experiments were performed at least 2 times with successful reproduction.
Randomization	Littermate mice were randomized between experimental groups by sex and cage; for experiments involving more than one litter, each litter

Randomization	was equivalently randomized between experimental groups. Experimental groups were age and sex matched as possible. Littermate controls were used for genetic experiments.
Blinding	Mice remained unblinded during experiments for the purpose of cohort assignment and experimental treatment. Mice were assigned a numeric ID which was used throughout data collection/analysis and matched to experimental group after data analysis. For imaging studies, brains were blinded by an independent researcher at the time of tissue freezing; slicing, staining, imaging, and image quantification were performed prior to unblinding.

Reporting for specific materials, systems and methods

We require information from authors about some types of materials, experimental systems and methods used in many studies. Here, indicate whether each material, system or method listed is relevant to your study. If you are not sure if a list item applies to your research, read the appropriate section before selecting a response.

Materials & experimental systems

n/a	Involved in the study
☐	☒ Antibodies
☒	☐ Eukaryotic cell lines
☒	☐ Palaeontology and archaeology
☐	☒ Animals and other organisms
☒	☐ Clinical data
☒	☐ Dual use research of concern
☒	☐ Plants

Methods

n/a	Involved in the study
☒	☐ ChIP-seq
☐	☒ Flow cytometry
☒	☐ MRI-based neuroimaging

Antibodies

Antibodies used

Primary antibodies used for murine imaging include chicken anti-GFP (Aves Labs GFP-1020, 1:200), rabbit anti-dsRed (Takara 632496, 1:300), chicken anti-GFAP (Invitrogen PA1-10004, 1:200 or 1:500), rat anti-GFAP (2.2B10, Invitrogen 13-0300, 1:200), rat anti-ER-TR7 (Novus Biologicals NB100-64932, 1:200), rabbit anti-aSMA (Abcam ab5694, 1:300), rat anti-CD31 (MEC13.3, Biolegend 102514, 1:200), goat anti-Desmin (GenWay Biotech GWB-EV0472, 1:200), rat anti-PDGFRb (APB5, Invitrogen 14-1402-82, 1:500), rabbit anti-NG2 (Millipore Sigma ab5320, 1:200), goat anti-Decorin (Novus Biologicals AF1060, 1:200), goat anti-Collagen 1 (Southern Biotech 1310-01, 1:500), rabbit anti-Collagen 6a1 (Novus Biologicals NB120-6588, 1:200 or 1:500), rat anti-Periostin (345613, Novus Biologicals MAB3548, 1:200), rat anti-ICAM1 (YN1/1.7.4, Biolegend 116110, 1:200), syrian hamster-anti-CD3e (500A2, BD Biosciences 553238, 1:200), goat anti-S100A8 (R&D Systems AF3059, 1:200), chicken anti-NeuN (Millipore Sigma ABN91, 1:200), rabbit anti-Iba1 (Aif3, Fujifilm Wako 019-19741, 1:200-1:1000), mouse anti-FGF13 (N235/22, Invitrogen MA5-27705, 1:100), goat anti-CD80 (R&D Systems AF740, 1:200), goat anti-ALPL (Novus Biologicals AF2910, 1:50), rabbit anti-LAMA1 (EPR27258-37, Abcam ab307542, 1:200), rabbit anti-SEMA3C (Invitrogen PA5-103168, 1:100), rabbit anti-CDH18 (Invitrogen PA5-112902, 1:50), rabbit anti-ALDH1A2 (Novus Biologicals NBP2-92915, 1:200), goat anti-SOX10 (R&D Systems AF2864, 1:300), rabbit anti-ASPA (Genetex GTX113389, 1:1000), mouse anti-E-Cadherin (Clone 36, BD Biosciences 610181), rat anti-I-A/I-E (MHCII, M5/114.15.2, eBioscience 14-5321-82), mouse anti-Ly76 (TER119, Biolegend 116232, 1:200), goat anti-mouse IgM (Invitrogen 31172, 1:200), and rabbit anti cleaved Caspase 3 (Cell Signaling Technology 9661T, 1:400; Cell Signaling Technology 9991, Histowiz). For marmoset imaging, rabbit anti-COL6 (Abcam ab6588, 1:500) was used.

Secondary antibodies were used at 1:500 (for thin sections) and 1:1000 (for thicker sections), as specified in relevant Methods sections. Secondary antibodies used include Donkey anti-rat IgG AF488 (Thermo Scientific A21208), Donkey anti-rat IgG AF555 (Thermo Scientific A78945), Donkey anti-rat IgG AF647 (Abcam ab150155), Donkey anti-rabbit IgG AF488 (Thermo Scientific A21206), Donkey anti-rabbit IgG AF555 (Thermo Scientific A31572), Donkey anti-rabbit IgG AF647 (Thermo Scientific A31573), Donkey anti-goat IgG AF488 (Thermo Scientific A11055), Donkey anti-goat IgG AF555 (Thermo Scientific A21432), Donkey anti-goat IgG AF647 (Thermo Scientific A21447), Donkey anti-chicken IgG AF488 (Sigma, SAB4600031-250UL), Donkey anti-chicken IgG AF647 (Thermo Scientific A78952), Goat anti-rat IgG AF488 (Thermo Scientific A11006), Goat anti-rabbit IgG AF555 (Thermo Scientific A21429), Goat anti-rabbit IgG AF647 (Thermo Scientific A21245), Goat anti-hamster IgG AF647 (Thermo Scientific A21451), Donkey anti-mouse IgG AF647 (Thermo Scientific A31571).

Antibodies used for flow cytometry include rabbit anti-Olig2 (Thermo Scientific P21954, 1:100), anti CD45 (30-F11, BD Biosciences 564279 or Biolegend 103132 or 103104, 1:400), anti-CD90.2 (Thy1, 53-2.1, Biolegend 140327, BD Biosciences 553004, 1:200), anti-CD11b (M1/70, Biolegend 101224 or BD Biosciences 563015, 1:400), anti-CD19 (6D5, Biolegend 115554, 1:400), anti-NK1.1 (PK136, Biolegend 108736, 1:200), anti-CD3e (17A2, Biolegend 100216, 1:200), anti-CD4 (RM4-5, Biolegend 100557, or GK1.5, BD Biosciences 563050, 1:200), anti-CD8[illegible] (53-6.7, Biolegend 100750, 1:200), anti-CD44 (IM7, Biolegend 103030, 1:200), anti-CD69 (H1.2F3, Biolegend 104505, 1:200), anti-CD62L (MEL-14, Biolegend 104407, 1:200), anti-Tbet (4B10, Biolegend 25-5825-80, 1:100), anti-Gata3 (TWAJ, eBioscience 12-9966-41, 1:100), anti-RORgt (B2D, eBioscience 17-6981-82, 1:100), anti-Ly6G (1A8, Biolegend 127624, 1:200), anti-IFNg (XMG1.2, Biolegend 505810, 1:100), anti-IL17A (TC11-18H10.1, Biolegend 506922, 1:100), anti-IL10 (JES5-16E3, eBioscience 12-7101-81, 1:100), anti-TCRgd (Biolegend 118118, 1:200 [extracellular] or 1:400 [intracellular]), anti-FoxP3 (eBioscience 53-5773-82, 1:100), anti-Ly6C (HK1.4, Biolegend 128011 or 128035, 1:400), anti-CD64 (X-54-5/7.1, Biolegend 139323 or BD Biosciences 558539, 1:200), anti-MERTK (DS5MMER, eBioscience 46-5751-80, 1:200), anti-CD9 (KMC8, BD Biosciences 564235, 1:200), anti-TREM2 (237920, R&D systems FAB17291A, 1:200), anti-CD63 (NVG-2, Biolegend 143904, 1:200), anti-I-A/I-E (MHCII, M5/114.15.2, BD Biosciences 748845, 1:400), anti-CD11c (N418, Biolegend 117339 or 117318, 1:200), anti-CD172a (SIRPa, P84, eBioscience 12-1721-80, 1:200), anti-Siglec-F (E50-2440, BD Biosciences 740956, 1:200), anti-Podoplanin (gp38, 8.1.1, Biolegend 127412, 1:200), anti-CD31 (390, Biolegend 102404 or 102408, 1:200), anti-EpCAM (G8.8, Biolegend 118230, 1:200), anti-PDGFRa

(APA5, Biolegend 135908, 1:200), anti-Sca-1 (Ly-6A/E, D7, Biolegend 108131, 1:200), anti-phosphoSMAD3 (EP823Y, Abcam ab52903, 1:50), and anti-CD16/32 (2.4G2, BD Biosciences 553142, 1:100 or 1:250).

Validation	All antibodies were validated by the manufacturer per species (mouse or marmoset) and per application (immunofluorescent imaging, flow cytometry).

Animals and other research organisms

Policy information about studies involving animals; ARRIVE guidelines recommended for reporting animal research, and Sex and Gender in Research

Laboratory animals

Mice (Mus musculus):

Fibroblast lineage tracing was performed using Col1a2creERT2 mice (MGI 6721050, from Bin Zhou, Institute of Biochemistry and Cell Biology, Shanghai Institutes for Biological Sciences) crossed with Rosa26TdT-Ai14 mice (R26-CAG-RFP, Jackson 007914) or with Rosa26Sun1GFP mice (Jackson 030952). For dMCAO experiments, a distinct Col1a2creERT allele was used (Jackson 029567). Additional stromal reporters used include Col1a1GFP mice (from David Brenner, University of California, San Diego); PdgfraGFP (PDGFRa-H2B-eGFP nuclear-localized GFP, Jackson 007669); and Rosa26Tdt-Ai14 mice crossed to Gli1creERT2 mice (Jackson 007913); Twist2cre mice (Jackson 008712); Acta2creERT2 mice; Ng2creER mice (Ng2creERTM, Jackson 008538); Atp13a5creERT2 mice; and Cthrc1creER mice (generously provided by Dean Sheppard).

Immune cell lineage tracing was performed using Rosa26TdT-Ai14 mice crossed to CD4cre mice (Jackson 022071); Ccr2creERT2 mice (from Burkhard Becher, University of Zurich, Switzerland); P2ry12creERT2 (Jackson 034727); Cx3cr1creER (Jackson 020940); and PF4cre (Jackson 008535). We also used T-bet (Tbx21)-zsGreen transgenic mice (generously provided by Jinfang Zhu, Lab of Immune System Biology, NIH). Cx3cr1creER mice were additionally crossed with Tgfb1GFP mice (MGI 3719583) and Tgfb1flox mice (Jackson 033001) to create in Cx3cr1creER; Tgfb1GFP/flox mice.

To generate Tgfbr2 conditional knockout mice, Col1a2creERT2 mice were crossed to Tgfbr2flox mice (both Tgfbr2-exon2flox, MGI 238451373, and Tgfbr2-exon4flox, Jackson 012603). To generate Cxcl12 conditional knockout mice, Col1a2creERT2 mice were crossed to Cxcl12flox mice (Jackson 021773). To generate Itgb8 conditional knockout mice, Itgb8flox mice (MGI 3608910) were crossed to Emx1cre mice (Jackson 005628) or hGfapcre mice (Jackson 004600); some hGfapcre mice were crossed to iSurecre (MGI 6361135) to optimize Cre efficiency. We also used Itgb8TdT mice (Itgb8-IRES-TdT, generously provided by Helena Paidassi). To generate myofibroblast deleter mice, Cthrc1creER mice were crossed to Rosa26DTA mice.

Mice were mixed gender animals backcrossed on C57BL/6 for at least 10 generations, or on a mixed genetic background (Gli1creERT2, Cx3cr1creER, Emx1cre). If not otherwise stated, all experiments were performed with 7-21 week old male and female mice. Controls are defined in relevant figure legends and in Methods. All mice were bred and maintained in specific-pathogen-free conditions, at 25°C and ambient humidity under a 12-hour day/night cycle, at the animal facilities of UCSF or UCSD.

Marmosets (Callithrix jacchus):

Outbred middle-aged marmoset monkeys (>5 years; median age ~7 years) were used in this study. No siblings were used. Animals were housed in family groups (12:12 hrs light/dark cycle, temperature 31°C, humidity 65%). Marmosets were obtained from the National Nonhuman Primate Breeding and Research Facility (Monash University, Australia).

Wild animals	No wild mice were used in this study.
Reporting on sex	Male and female mice were used for all experiments and data was analyzed by sex; as no sex-specific trends emerged, data is not reported separately by sex.
Field-collected samples	No field collected samples were used in this study.
Ethics oversight	All mice were bred and maintained in specific-pathogen-free conditions at the animal facilities of UCSF or UCSD and were used in accordance with institutional guidelines and under study protocols approved by the UCSF or UCSD Institutional Animal Care and Use Committee (protocols AN193180-01J, AN195716-01B [UCSF], s14044 [UCSD]). Marmoset experiments were conducted according to the Australian Code of Practice for the Care and Use of Animals for Scientific Purposes and were approved by the Monash University Animal Ethics Committee.

Note that full information on the approval of the study protocol must also be provided in the manuscript.

Plants

Seed stocks: Not applicable

Novel plant genotypes: Not applicable

Authentication: Not applicable

Flow Cytometry

Plots

Confirm that:

☒ The axis labels state the marker and fluorochrome used (e.g. CD4-FITC).

☒ The axis scales are clearly visible. Include numbers along axes only for bottom left plot of group (a 'group' is an analysis of identical markers).

☒ All plots are contour plots with outliers or pseudocolor plots.

☒ A numerical value for number of cells or percentage (with statistics) is provided.

Methodology

Sample preparation

Flow cytometry preparation:

Single cell suspensions were prepared from tissues including brain, spinal cord, meninges, blood, and spleen. Immediately following CO2 euthanasia, spleens were removed into RPMI/10% FBS and peripheral blood was collected through the right ventricle into heparin tubes. Mice were subsequently transcardially perfused through the left ventricle with 10mL of DPBS, decapitated, and brains were carefully removed from skullcaps and placed in iMED+ as above. For select experiments, spinal cords were carefully dissected from vertebra. Cortex, lesion, and meninges were dissected as above. Brain was weighed and subsequently homogenized in iMED+ homogenized using a 2mL glass tissue grinder (VWR; 6 plunges, followed by filtration through a 70um filter, addition of 2mL iMED+, and 6 more plunges). Filtered suspensions were centrifuged at 220g for 10 minutes and resuspended in 5mL of 22% Percoll (GE Healthcare) in Myelin Gradient Buffer (5.6mM NaH2PO4•H2O, 20mM Na2HPO4•2H2O, 140mM NaCl, 5.4mM KCl, 11mM glucose in H2O). 1mL of PBS was layered on top of Percoll. Samples were centrifuged at 950g for 20 minutes at 4C with no break to separate myelin and resuspended in FACS buffer. Dissected meninges were incubated in digestion medium (RPMI/10% FBS/80ug/mL DNase I/40ug/mL Liberase TM (Roche)). Tissue was subsequently mashed through 70um filters, followed by centrifugation and resuspension in FACS buffer. Spleens were prepared by mashing tissue through 70um filters without tissue digestion, followed by centrifugation. Red blood cells were lysed for 2 minutes using 1X Pharm-Lyse and the remaining cell pellet were resuspended in FACS buffer.

Blood samples were centrifuged for 5 minutes at 500g. Pellets were resuspended in 1X Pharm-Lyse 5 minutes at room temperature, followed by centrifugation and suspension in FACS buffer. For cytokine restimulation assays, samples were transferred to U-bottom plates and incubated in stimulation medium (RPMI supplemented with 10% FBS, 1% penicillin/streptomycin, 1X Glutamax (Thermo Scientific), 1X HEPES buffer (Fisher), 1X non-essential Amino Acids (Thermo Scientific), 1mM NaC3H3O3 (Thermo Scientific), 55�M b-mercaptoethanol, 1X Cell Stimulation Cocktail (Tonbo), and 1X Brefeldin A (Thermo Scientific)) at 37�C for 3 hours, followed by centrifugation and transfer to a V-bottom plate.

Resuspended samples were stained in 96-well V-bottom plates. Surface staining was performed at 4C for 45 minutes in 50uL staining volume. For experiments involving intra-cellular staining, cells were fixed and permeabilized using Foxp3 Transcription Factor Staining Buffer Set (eBioscience) followed by staining at 4C for 1 hour in 50uL staining volume.

Nuclear preparation (mouse):

For nuclear flow cytometry and single nuclear RNAseq experiment 1 (timecourse), dura, lesion, and perilesional cortex were microdissected and processed separately. For RNAseq experiment 2 (WT, cKO, and ADWA11), only lesions were dissected. Microdissection involved meningeal/skullcap separation, removal of subcortical structures, and separation of lesions from skullcaps (lesions often separate from cortex during initial dissection but can be micro-dissected as necessary). For snRNAseq, tissue from male and female mice within experimental conditions was combined. Tissue was processed using ST-based buffer protocol, with the following modifications: initial centrifugation was performed at 500g for 10 minutes. After lysis/initial centrifugation, nuclei were resuspended in 1mL ST buffer (nuclear flow cytometry, RNAseq experiment 2) or PBS/1% BSA/0.2UuL Protector RNAse inhibitor (Roche) (RNAseq experiment 1), filtered through 35um cell strainers, and subsequently processed as below.

For nuclear flow cytometry and single nuclear RNAseq experiment 2, nuclei were centrifuged for 5 minutes at 500g, resuspended in FANS buffer (DPBS/1% BSA/0.1mM EDTA80) with 2ug/uL DAPI and 0.2U/uL RNase inhibitor (snRNAseq), and stained (nuclear flow) or sorted (snRNAseq). For single nuclear RNAseq experiment 1, cell counts were performed after initial

centrifugation (NucleoCounter, Chemometic), and a maximum of 2x106 nuclei were multiplexed using CellPlex Multiplexing technology (10X Genomics) according to the manufacturer's instructions (using protocol 1 for nuclear multiplexing, with only one wash after multiplexing to increase yield). Nuclei were resuspended in FANS buffer with 0.2U/uL RNase inhibitor and 2ug/uL DAPI and nuclear concentrations were determined. Immediately before sorting, multiplexed microanatomical regions (including lesion, parenchyma, and dural meninges) from individual mice were combined at desired ratios (75% lesion, 25% parenchyma).

Nuclear preparation (marmoset):

For single-nuclei RNA sequencing (snRNAseq), naïve control marmosets (n=3; 1 female, 2 male; median age 4Y) were administered an overdose of pentobarbitone sodium (100 mg.kg-1; intraperitoneal). Following apnea, frontal lobes were recovered and dissected under aseptic conditions in sterile ice-cold phosphate buffered saline (PBS; 0.1M; pH 7.2). Tissues were and snap frozen in isopentane chilled in liquid nitrogen. The procedures/ dissections were performed in chilled RNAase-free PBS with RNase-free sterilized instruments under RNase-free conditions. Approximate time from apnea to snap-freezing ranged from 20-30 minutes. All 6 samples passed QC. For nuclear isolation, frozen cerebral tissues from the injured cohort were finely pulverized to powder in liquid nitrogen, and 50 mg of pulverized tissue was added into 5 mL of ice-cold lysis buffer (320mM sucrose, 5mM CaCl2, 3 mM Mg(Ace)2, 10mM Tris-HCl [pH 8], protease inhibitors without EDTA, 0.1 mM EDTA , RNAse inhibitor [80U/mL], 1mM DTT, 0.1% TX-100 [v/v]). The suspension was homogenized in a Dounce tissue grinder (15mL, RNAse free, ice-cold) using loose and tight pestles, 30 cycles each, with constant pressure and without introduction of air. The homogenate was strained (40µm) and the strainer was washed with isolation buffer (1800 mM sucrose, 3mM Mg(Ace)2, 10mM Tris-HCl [pH 8], protease inhibitors without EDTA, RNAse inhibitor [80U/mL], 1mM DTT). The suspension was mixed via tube inversion and then gently pipetted onto the isolation buffer cushion (5 mL) without disrupting the phases and centrifuged at 30000g for 60 min at 4℃. The resultant supernatant was removed and 100µl of resuspension buffer (250mM sucrose, 25mM KCl, 5mM MgCl2, 20mM Tris-HCl [pH 7.5], protease inhibitors without EDTA, RNAse inhibitor [80U/mL], 1mM DTT) was added dropwise on the pellet and incubated on ice for 15 minutes. Pellets were resuspended, pooled, and filtered through 40um cell strainers. Finally, nuclei were counted and diluted to 1 million/mL with sample-run buffer (0.1% BSA, RNAse inhibitor [80U/mL], 1mM DTT in DPBS).

Instrument

Flow cytometry was performed on a BD LSRII Fortessa Dual; flow cytometric sorting was performed on a BD FACSAria.

Software

Flow cytometric data was acquired using FACSDiva v9.0 (BD Biosciences) and analyzed using FlowJo v10.7.2 (BD Biosciences)

Cell population abundance

For each experiment, single-cell suspensions were incubated with viability dyes, with live cells gated as viability-dye-negative. Cell counts were performed using flow cytometry counting beads (CountBright Absolute; Life Technologies) per manufacturer's instructions. Populations were given as percentage of live CD45+ cells or as backcalculated cell numbers.

Gating strategy

Oligodendrocyte-lineage nuclei were identified as DAPI+, Pdgfr[illegible]GFP-hi, Olig2+. Fibroblast nuclei were identified as DAPI+, PdgfraGFP-int or DAPI+, Col1a2creER; Rosa26Sun1GFP+ (snRNAseq experiment 1, resting dural meninges). Bulk nuclei were identified as DAPI+. Global lymphocytes were defined as CD45+, Thy1+. T cells were identified as CD45+, CD11b-, CD19-, NK1.1-, CD3e+, CD4+ (CD4 T cells; further subset as FoxP3+ [regulatory T cells] or FoxP3- [conventional CD4 T cells]), CD8a+ (CD8 T cells), or TCRgd+ (gdT cells) and were further defined as CD44+, CD69+ (resident memory T cells, TRM), CD62L+ (naïve T cells), CD62L-, CD44+ (activated T cells), or CTVdiluted (proliferating T cells). Additionally, CD4 T cells were defined as T-bet+ (Th1 T cells), Gata3+ (Th2 T cells), or RORgt+ (Th17 T cells), and various T cell subsets were defined as cytokine-positive or negative (IFNg, IL17A, or IL10). Neutrophils were defined as CD45+, CD11b+, Ly6G+ (and optionally Thy1-, CD19-, NK1.1-). Monocytes were defined as CD45+, CD11b+, Ly6G-, Ly6C+ (and optionally Thy1-, CD19-, NK1.1-, Siglec F-). Microglia were defined as CD45int, CD11b+. Macrophages were defined as CD45+, Ly6G-, Ly6G-, CD64+ (optionally MERTK+). Microglia/ macrophages were further defined as Damage Associated Microglia/Scar Associated Macrophages (DAM/SAM, CD9+ and CD63+/TREM2+). cDCs were identified as CD45+, Ly6G-, Ly6C-, CD64-, MHCII+, CD11c+, and were further defined as cDC1s (CD11blo, optionally SIRPa-) or cDC2s (CD11bhi, optionally SIRPa+). B cells were defined as CD45+, Thy1-, CD19+. Eosinophils were defined as CD45+, Thy1-, CD19-, NK1.1-, Ly6G-, CD11b+, Siglec F+. Populations were back-gated to verify purity and gating.

☒ Tick this box to confirm that a figure exemplifying the gating strategy is provided in the Supplementary Information.
